# Domestic dog attacks on livestock referred to a Veterinary Teaching Hospital

**DOI:** 10.3389/fvets.2024.1342258

**Published:** 2024-02-21

**Authors:** Mariana da Costa Gonzaga, José Renato Junqueira Borges, Teresa Souza Alves, Davi Emanuel Ribeiro de Sousa, Márcio Botelho de Castro, Antonio Carlos Lopes Câmara

**Affiliations:** ^1^Large Animal Veterinary Teaching Hospital, College of Agronomy and Veterinary Medicine, Universidade de Brasília, Brasília, Federal District, Brazil; ^2^Veterinary Pathology Laboratory, College of Agronomy and Veterinary Medicine, Universidade de Brasília, Brasília, Federal District, Brazil

**Keywords:** dog bites, farm animals, horses, wounds, ruminants, pigs

## Abstract

Losses and the economic impact of dog attacks (DAs) on livestock are often overlooked and include factors such as decreased production, expenses for treatment and handling, and death of injured animals. This study evaluated the epidemiological, therapeutic, and pathological findings of DAs on livestock over an 11-year survey using the records of farm animals referred to a Veterinary Teaching Hospital. Livestock attacked by dogs included 31 sheep, 11 horses, 3 cattle, 3 goats, and 2 pigs, totaling 50 animals. Anatomical locations injured by dog bites were identified as head/neck, thoracic/pelvic limbs, abdomen/flank, rump/tail, and multiple affected regions (two or more bite sites). Additionally, the severity of the injuries was classified into four degrees adapted from the classification of dog bite injuries in children. Most livestock presented Grade 1 (26%) and Grade 2 (28%) injuries, while Grade 3 and Grade 4 injuries were observed in 46% of DAs. Furthermore, 35 animals (70%) were discharged, 9 (18%) died, and 6 (12%) were humanely euthanized. DAs may represent a significant cause for referring livestock species to clinical care, severe injuries, and a considerable number of deaths. In this study, we provide information regarding DAs on livestock for the first time in Midwestern Brazil.

## 1 Introduction

Brazil is a major player in the global meat trade, with its most significant livestock production located in the Midwestern region. Understanding and preventing diseases and other issues that negatively impact livestock production is crucial, as they can cause substantial economic losses. While the incidence of dog bites affecting humans is not well-documented globally (1), dog attacks (DAs) are considered a common public health concern (2) and have also been reported in wildlife and livestock (3, 4). Stray dogs can severely affect wild animals by transmitting diseases, competing for food, and causing aggression, predation, displacement, and death (4). DAs are also a concern for livestock animals, such as cattle, goats, sheep, and horses (5–9). Wild predators affect livestock production and are the subject of human–wildlife conflicts worldwide (10).

DAs commonly result in single or multiple traumatic injuries to the skin and soft tissues, such as blunt trauma, abrasions, bruises, cuts, and lacerations. Affected animals may also experience bone fractures, severe blood loss, and damage to vital organs (5, 7, 11, 12), and acquire infectious diseases, such as rabies and secondary bacterial infections at the bite sites as well (13, 14).

Losses and the economic impact of dog bites on livestock are often overlooked and include factors such as decreased production, expenses for treatment and handling, and death of injured animals. This study evaluated the epidemiological, therapeutic, and pathological findings of DAs on livestock over an 11-year survey using the records of farm animals referred to a Veterinary Teaching Hospital.

## 2 Materials and methods

An 11-year (2010–2020) survey was conducted on the records of DAs on livestock at the Large Animal Veterinary Teaching Hospital, University of Brasília, Midwestern Brazil. Retrieved data included epidemiological findings (species, gender, age, month of occurrence), treatments (topical and/or systemic therapy), and outcomes (hospital discharge, death, or euthanasia *in extremis*). We calculated the annual average of farm animals raised in the Federal District (AAF) between 2016 and 2020 from the records provided by the Federal District Department of Agriculture.

DA diagnosis was performed based on the clinical history (cases witnessed by the farmers or their foremen) and the pattern of external and internal injuries (15, 16). Anatomical locations injured by dog bites were identified as head/neck, thoracic/pelvic limbs, abdomen/flank, rump/tail, and multiple affected regions (two or more bite sites). Additionally, the severity of the injuries was classified into four degrees adapted from the classification of dog bite injuries in children (17) as follows: Grade 1 – superficial injury without the involvement of muscle; Grade 2 – deep injury with muscle damage; Grade 3 – deep injury with the involvement of muscle and tissue defect; Grade 4 – Grade 3 combined with vascular, nerve and/or bone injury, and/or internal organ involvement. Therapeutic options recorded for DAs on livestock were retrieved (clinical or surgical treatment), and euthanized animals were excluded due to not receiving any therapy.

Fisher’s test or a Chi-square test (performed with GraphPad Prism 8.01 software) was used to compare the frequencies of species, anatomical locations, month of occurrence, injury grade, and lethality in animals affected by DAs.

## 3 Results

**Figure 1:**
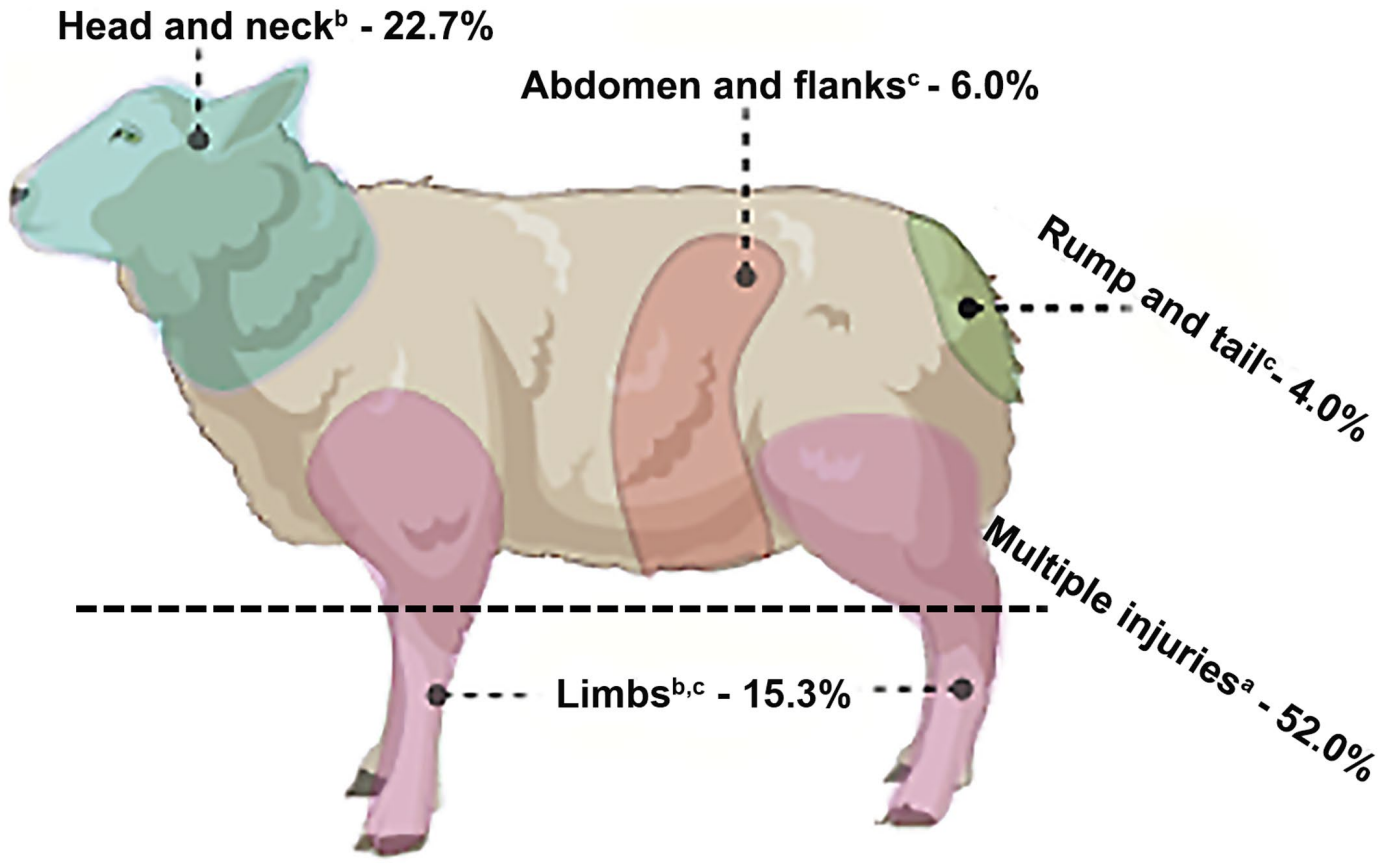
Frequencies (%) of dog bite sites in livestock affected by DAs in Midwestern Brazil. Different lowercase letters are significant (*p* < 0.05).

**Figure 2:**
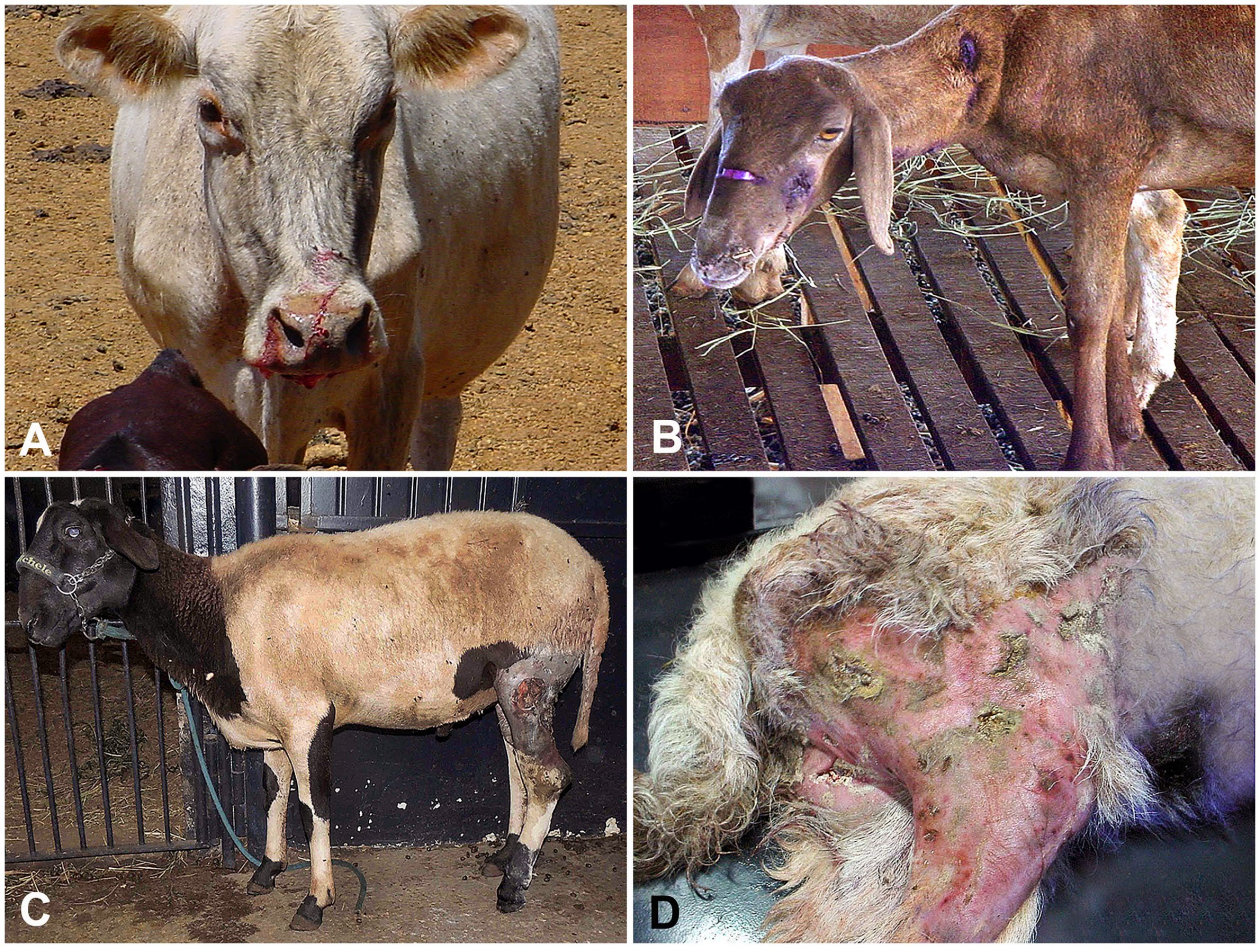
Livestock affected by DAs. **(A,B)** Mixed-breed cow and an ewe with superficial skin injuries (Grade 1), respectively. **(C,D)** Ram **(C)** and a doe **(D)** with dog bites affecting superficial muscle tissues (Grade 2), respectively.

**Figure 3:**
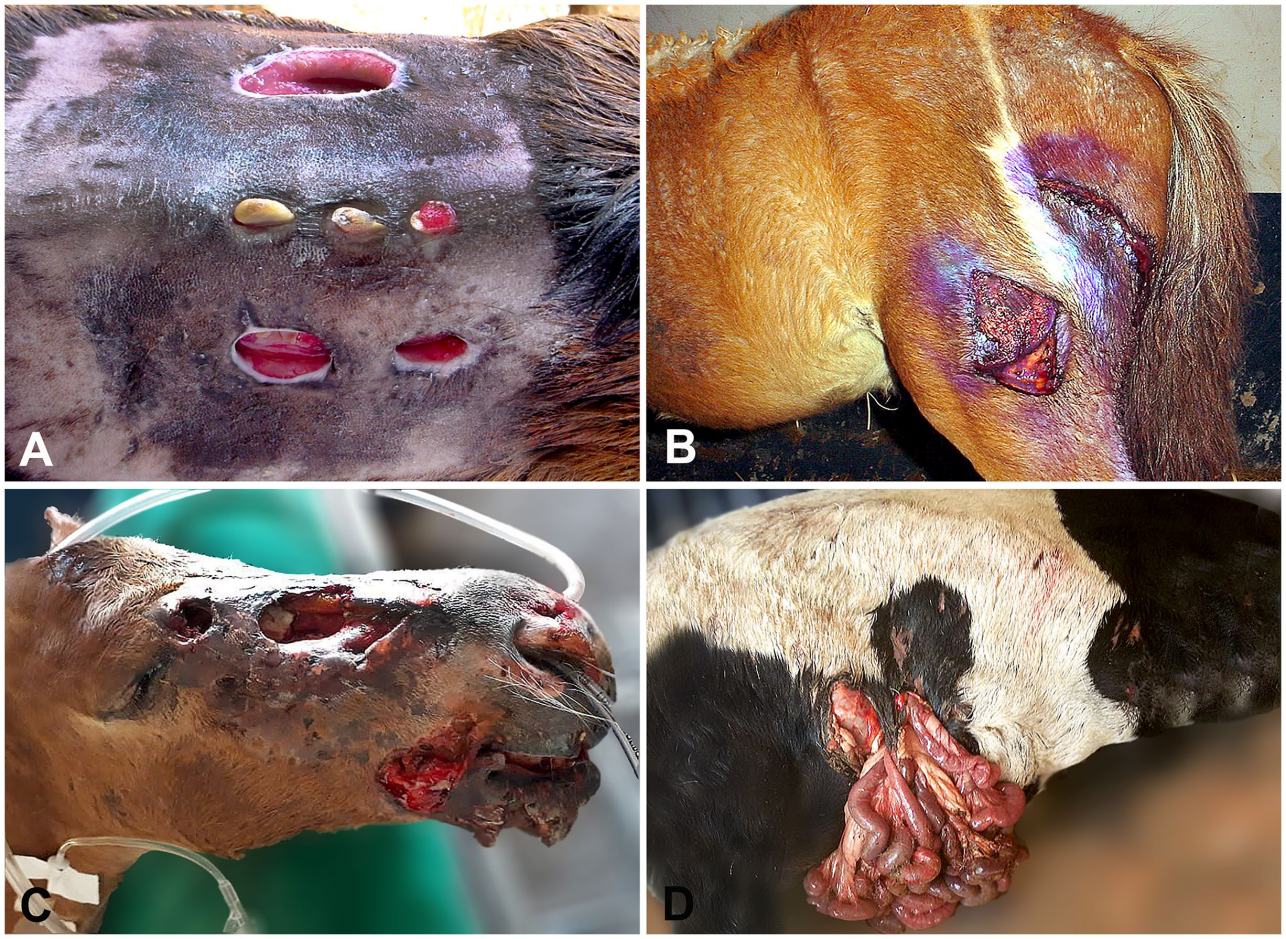
Livestock affected by DAs. **(A,B)** An ewe and a foal with skin injuries and deep muscle lacerations (Grade 3), respectively. **(C,D)** A foal and an ewe with severe dog bite injuries in the head and evisceration (Grade 4), respectively.

The total number of referred animals (RAs), the annual average of farm animals raised in the Federal District (AAF), DAs on livestock in the Federal District between 2016 and 2020, the classification of DA injuries, and the lethality are shown in Table 1. DAs were witnessed by the farmers or their foremen in 84% of the cases (*n* = 42/50), with 23 (54.7%) of these incidents infringed by a single dog and 19 (45.3%) by two or more dogs. When recorded (*n* = 26 cases), most aggressor animals were identified as medium or large stray mixed-breed dogs (*n* = 20; 76.9%), Brazilian mastiffs (*n* = 2; 7.7%), Rottweilers (*n* = 2; 7.7%), and pitbulls (*n* = 2; 7.7%). The body location of DAs in all livestock is shown in Figure 1. The livestock attacked by dogs included 31 sheep, 11 horses, 3 cattle, 3 goats, and 2 pigs, totaling 50 animals. While female animals were most affected by DAs (70%, *n* = 35), male animals accounted for 30% (*n* = 15) of the cases (*p* < 0.05), ranging from 1 day to 7 years of age. DAs on livestock occurred throughout the year, with the highest incidence in November (*n* = 8) and the lowest in February (*n* = 1) and September (*n* = 1) (*p* < 0.05) in Midwestern Brazil. There were no significant differences in the frequency of DAs between all other months (*p* > 0.05). Most livestock presented Grade 1 (26%) (Figures 2A,B) and Grade 2 (28%) injuries (Figures 2C,D), while Grade 3 (Figures 3A,B) and Grade 4 injuries were observed in 46% (23/50) of DAs (Figures 3C,D).

**Table 1:** Total of referred animals (RAs) and animals affected by dog attacks (DAs), annual average of farm animals raised in the Federal District area (AAF), and standard deviation in the Federal District between 2016 and 2020, classification of DA injuries, and lethality in an 11-year survey at the Veterinary Teaching Hospital in Midwestern Brazil.

Species	RAs	AAF	DAs	Grade of injuries				Lethality
		m ± sd (2016–2020)	*n*/incidence/frequency (%)	1	2	3	4	
Horse	2,435 (54.1%)	18,152.0 ± 871.9	11 (0.4%^b^/22.9%^b^)	2 (18.1%)	3 (27.3%)	3 (27.3%)	3 (27.3%)	4 (36.3%)
Sheep	811 (18.1%)	21,015.5 ± 2,574.6	31 (3.8%^a^/64.6%^a^)	10 (32.3%)	7 (22.6%)	8 (25.8%)	6 (19.3%)	8 (25.8%)
Cattle	809 (18.0%)	86,729.7 ± 4,211.8	3 (0.4%^b^/6.2%^c^)	1 (33,3%)	2 (67,7%)	–	–	0
Swine	144 (3.2%)	145,869.2 ± 72,775.6	2 (1.4%^ab^/4.2%^c^)	–	2 (100%)	–	–	0
Goat	232 (5.2%)	3,267.2 ± 354.5	3 (1.3^b^/6.0%^c^)	–	–	–	3 (100%)	3 (100%)
Mule	51 (1.1%)	1,341.6 ± 87.8	–	–	–	–		–
Donkey	15 (0.3%)	279.6 ± 20.1	–	–	–	-		–
Total	4,497	305,759.4 ± 23,482.8	50 (1.1%)	13 (26.0%)	14 (28.0%)	11 (22.0%)	12 (24.0%)	15 (30.0%)

AAF: medium (m) and standard deviation (sd). DAs: n = number of affected animals; incidence = DAs (%) in the total of referred animals; frequency: affected animals (%) in the total of DAs. Different letters in the same column are significant (*P* < 0.05).

**Figure 4:**
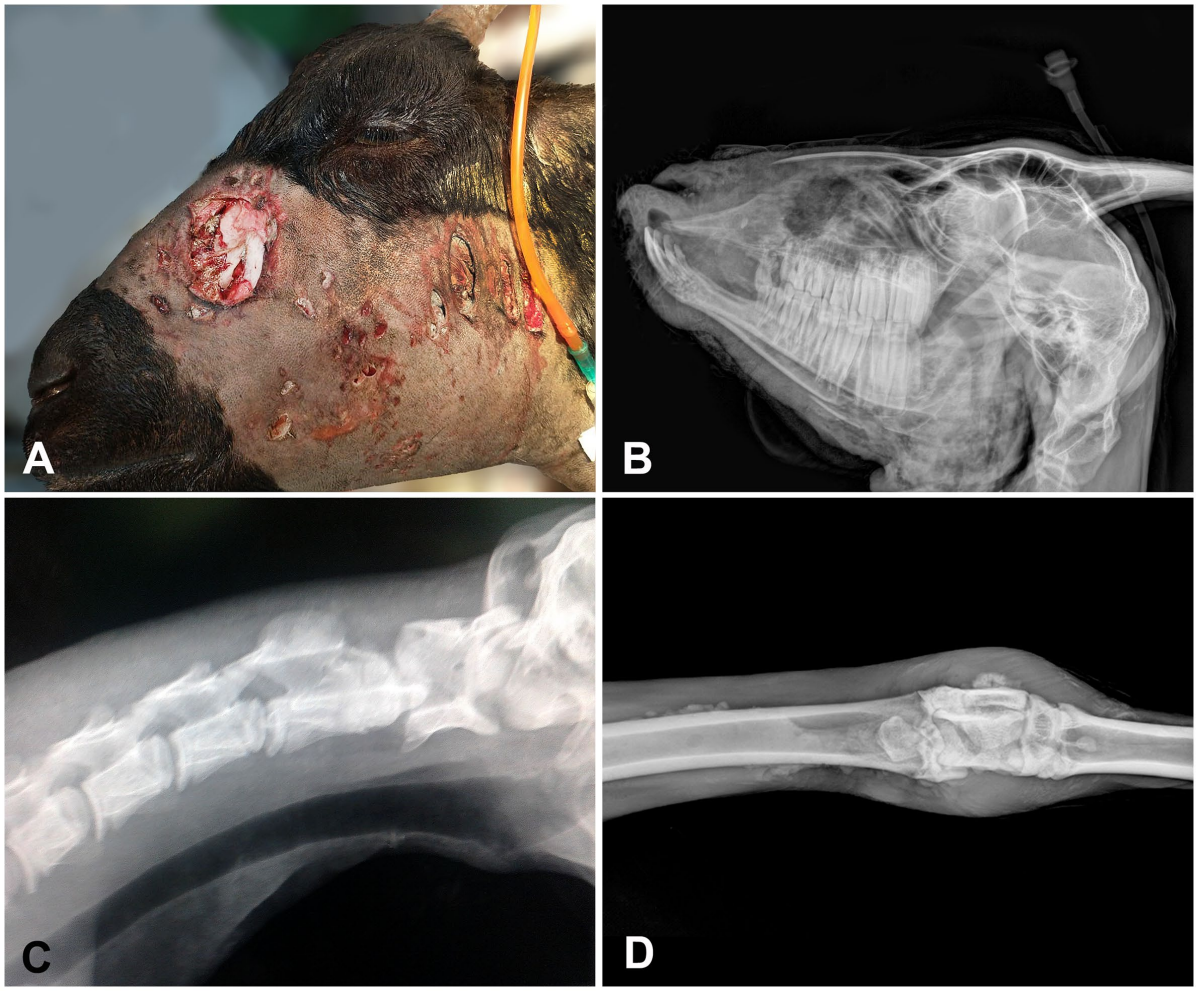
Livestock affected by DAs. **(A)** A doe with several deep injuries and fracture of the nasal bone. **(B)** Latero-lateral radiograph of the doe on **(A)** presenting fracture on the nasal bone and mandible. **(C)** Latero-lateral radiograph presenting dislocation of the first and second cervical vertebrae in a sheep. **(D)** Dorso-plantar radiograph showing osteomyelitis in the metatarsal joint of a goat.

Furthermore, 35 animals (70%) were discharged, 9 (18%) died, and 6 (12%) were submitted to euthanasia *in extremis*. Euthanized animals presented extensive and severe injuries, including abdominal laceration with evisceration in two sheep (Figure 3D), facial fractures and trachea laceration (one pony and one goat) (Figures 4A,B), dislocation of the first and second cervical vertebrae (one sheep) (Figure 4C), and osteomyelitis (one goat) (Figure 4D).

Regardless of the location of lesions and the degree of injuries, surviving animals had a recovery time ranging from 2 to 135 days (an average of 36 days). The hospitalization time was from 1 to 20 days (with an average of 4 days) in fatal DAs or elected euthanasia cases. An average hospital stay of 24 days (10 to 31 days) was observed in animals with superficial DA lesions (Grade 1). Affected animals with muscle damage and deep injuries (Grades 2 and 3) showed an average hospital stay of 44 days (with stays ranging from 2 to 83 days). The average length of stay in a hospital for livestock with Grade 4 injuries was approximately 67 days (with stays ranging from 25 to 138 days).

Regarding therapeutic options, only 44 animals were included in the study since 6 livestock were euthanized without treatment. Most animals (*n* = 37; 84.1%) were treated clinically (broad-spectrum antibiotics, anti-inflammatory drugs, and topical wound dressing), and wound healing was achieved by secondary intention. Surgical treatment was performed on seven animals (15.9%). Three cases with acute injuries were vigorously cleaned and sutured, and healing was accomplished by primary intention. Other surgical procedures include limb amputation (*n* = 1), tail docking (*n* = 1), reconstructive nasal surgery (*n* = 1), and mandible osteosynthesis (*n* = 1).

## 4 Discussion

Dog bites have become a significant concern for both humans and domestic animals since the domestication of dogs. One of the most notable examples is the increase in dog attacks on sheep, which result in significant losses of herds (18). DAs have been related to the instinct of dogs to attack other animals that they associate with prey (19, 20).

In this study, sheep were the most affected species by dog bites, considering the referred animals for clinical care and the annual average of livestock raised in the region, which is in line with the previous reports in other countries (5, 9, 12, 21–24). Attacks by wild dogs may result in the deaths of an average of 1–9% of animals in sheep flocks in the Western USA (25). Additionally, domestic dogs are the second most important cause of animal injury in livestock in Kansas State, USA (5) and are also one of the most relevant causes of sheep losses in some regions of Australia (26). In Italy, horses and cattle were the second and the third most affected species by wild predators, respectively (27), as observed in domestic DAs on livestock in this study. Cattle were the second most affected species by wild and stray dogs in Eastern New South Wales, Australia (23).

Although wild predators are a significant cause of loss in livestock, domestic DAs may significantly affect sheep flocks in some locations (22, 23). Sheep have historically been perceived as a natural prey species for a wide range of wild predators; however, domestic dogs are currently the primary culprits responsible for most attacks on flocks (28). Even though sheep have been the most affected species, differences in the species composition of livestock herds in a region could likely influence the frequency of DAs. The abundance of livestock seems to be a better indicator for predicting DAs in an area compared to the local dog abundance (29).

It is important to note that wild predators such as wolves, coyotes, and bears are not present in South America in the area of this study. Brazilian native wild canids do not attack livestock, and the injuries detected in affected animals showed no hallmarks supporting attacks by big wild cats (puma and jaguars) (30, 31). In addition, most cases of DAs recorded were witnessed, while other cases evidenced injuries morphologically compatible with dog bites (15, 16). Moreover, stray dogs are frequently detected in rural and wild areas of Brazil (32, 33). Recently, a method for canine DNA identification in dog bite injuries has been proposed for forensic death identification in cats (15). Unfortunately, DNA tests and stored samples collected from dog bite injuries were unavailable, which would have undoubtedly strengthened the diagnosis of DAs in this study. Even though most previous studies on DAs on livestock were supported by epidemiological data and morphological features of dog bite injuries (5, 9, 12, 21–24), a protocol of investigation, including tests for canine DNA identification in dog bite injuries, will certainly contribute to further studies in the field.

Females animals (70%) were most affected by DAs in our study. Identification of the sex of the animals has not been determined in surveys of DAs on livestock worldwide (12, 21–24, 27). Considering commercial herds of livestock, the number of breeding female animals is generally much higher than that of breeding male animals. Rams usually have a proportion of approximately 1:30 ewes in the flocks (34) and may justify the most affected species observed in this study.

Multiple dog bites scattered throughout the body were the most common injury features in the affected animals in this study. This particular pattern likely occurred as a result of an animal being knocked to the ground by a predator, which then proceeds to inflict multiple bites (5, 7, 8), or when various dogs attack a single animal or several animals simultaneously (26). In Alberta, Canada, DAs on livestock frequently showed bite marks on the head, neck, hind limbs, flank, internal organ damage, and tears in the hide and ears (11), as detected in most animals in our study. The attack of dog packs may have a potentially harmful effect by inflicting severe and extensive tissue damage (35). The social facilitation of predatory sheep-chasing behavior in packs of dogs (36) could lead to multiple tissue injuries. Domestic dogs usually do not chase or kill livestock for food and may attack indiscriminately, resulting in various sites of injury (7, 8).

When DAs affected a single anatomical region of the body, the head and the neck were the most commonly bite-injured locations in this survey, as previously reported in coyote attacks on livestock (8). Dog bites were also a relevant cause of limb injuries and amputations in sheep and goat flocks in a survey performed in a Veterinary Medical Teaching Hospital in California, USA (37). As observed in this study, domestic dogs usually attack any part of their prey’s body (8, 11).

We observed that most DAs on farm animals resulted in Grade 2 and Grade 3 injuries (17) characterized by deep muscle, vascular, and bone tissue damage with or without the internal organ involvement. Although a standard classification for DA injuries in livestock is still lacking worldwide, different degrees of injury, such as severe internal organ damage, evisceration, eventration, deep and superficial bleeding, tears in the hide and ears, and scratches, have been reported (7, 11).

The hospitalization time for livestock with DAs varies according to the number and severity of the injuries. Animals that suffered lesions in multiple regions had an average hospitalization time of approximately 15 days, while patients with lesions in just one location had almost four times fewer hospitalization days (a mean of 4 days). Even with treatment, the mortality rate (15/50, 30%) was considered high. Generally, dog attacks on animals, especially those with gregarious behavior, tend not to result in the immediate death of the injured animal. Instead, the vast majority succumb later due to severe and extensive injuries and secondary bacterial infections (6, 12, 20). As previously reported (3, 12, 20), our study also evidenced the potential of DAs in promoting economic losses in livestock production due to treatment expenses and animal deaths resulting from attacks.

The management and therapy of DA wounds in Midwestern Brazil were mainly based on cleaning, administering topical antimicrobial prophylaxis, and resorting to surgical intervention in some cases, taking into account that there is no specific treatment and management for dog attacks in domestic animals (14) nor any consensus in their therapeutic protocols (38). Antibiotics are usually reliable therapeutics because dog bites promote contaminated wounds (17, 39). In severe DA injuries (12, 39), as observed in some animals in this study, surgical procedures (17) and amputation were performed (37), as necessary.

## 5 Conclusion

Considering all the limitations of this study, DAs may represent a significant cause for referring livestock species to clinical care, severe injuries, and a considerable number of deaths. We provide information regarding DAs on livestock for the first time in Midwestern Brazil, one of the world’s largest animal protein-producing regions. In Brazil, DAs are possibly underreported since the most significant commercial herds of beef cattle and sheep are raised extensively on vast areas of pasture. Further broad studies are required to determine the real impact of DAs on livestock production in Brazil. Additionally, effective management and measures to mitigate economic losses due to DAs remain to be determined in the herds in the region.

## Data availability statement

The original contributions presented in the study are included in the article/supplementary material, further inquiries can be directed to the corresponding author.

## Ethics statement

The requirement for ethical approval was waived by the Ethics Committee on the Use of Animals (CEUA) of the Faculty of Agronomy and Veterinary Medicine of the University of Brasília (Brasília, Brazil) for the studies involving animals because the livestock included in this study were under care at the Large Animal Veterinary Teaching Hospital, Universidade de Brasília, Brasília-DF, Brazil. The owners signed a consent form to permit hospitalization, surgery, and treatment. Additional consent was obtained for using the images for research purposes. The authors also confirm that the study has followed the guidelines of the 1964 Declaration of Helsinki and its later amendments. The studies were conducted in accordance with the local legislation and institutional requirements. Written informed consent was obtained from the owners for the participation of their animals in this study.

## Author contributions

MG: Data curation, Formal analysis, Investigation, Methodology, Writing – original draft. JB: Conceptualization, Methodology, Supervision, Writing – original draft. TA: Data curation, Formal analysis, Investigation, Writing – original draft. DS: Methodology, Data curation, Writing – review & editing. MC: Formal analysis, Investigation, Conceptualization, Methodology, Writing – review & editing. AC: Conceptualization, Methodology, Writing – review & editing, Funding acquisition, Supervision.
